# Ru(II) Complexes Bearing O, O-Chelated Ligands Induced Apoptosis in A549 Cells through the Mitochondrial Apoptotic Pathway

**DOI:** 10.1155/2020/8890950

**Published:** 2020-08-17

**Authors:** Jincan Chen, Jie Wang, Yuanyuan Deng, Tao Wang, Tifang Miao, Chengpeng Li, Xianhong Cai, Ying Liu, Justin Henri, Lanmei Chen

**Affiliations:** ^1^Guangdong Key Laboratory for Research and Development of Nature Drugs, Marine Biomedical Research Institute, School of Pharmacy, Guangdong Medical University, Zhanjiang 524023, China; ^2^School of Nursing, Zhengzhou University, Zhengzhou 450001, China; ^3^Centre for Molecular Medicine and Innovative Therapeutics, Murdoch University, Perth 6150, Australia; ^4^School of Chemistry and Materials Science, Huaibei Normal University, Huaibei 235000, China; ^5^The Public Service Platform of South China Sea for R&D Marine Biomedicine Resources, Marine Biomedical Research Institute, Guangdong Medical University, Zhanjiang 524023, China; ^6^School of Medicine Deakin University, Geelong, Victoria 3128, Australia

## Abstract

Two new Ru(II) complexes containing O, O-chelated ligands, Ru(dip)_2_(SA) (**Ru-1**) and Ru(dmp)_2_(SA) (**Ru-2**) (dip = 4,7-diphenyl-1,10-phenanthroline; dmp = 2,9-dimethyl-1,10-phenanthroline; SA = salicylate) were synthesized to evaluate their cytotoxicity *in vitro*. These complexes were found to exhibit moderate antitumor activity to different types of human cancers, including A549 (human lung carcinoma), MCF-7 (breast cancer), HeLa (human cervical cancer), and HepG2 (human hepatocellular carcinoma) cell lines, but displayed low toxicity to human normal cell lines BEAS-2B (immortalized human bronchial epithelial cells) when compared with that of cisplatin. Further studies revealed that these complexes could induce apoptosis in A549 cells, including activating caspase family proteins and poly (ADP-ribose) polymerase (PARP), reducing Bcl-2/Bax and Bcl-xl/Bad ratio, enhancing cellular reactive oxygen species (ROS) accumulation, triggering DNA damage, decreasing mitochondrial membrane potential (MMP), and leading cytochrome *c* release from mitochondria. Notably, complex **Ru-1** showed low toxicity to developing zebrafish embryos. The obtained results suggest that these new synthetic complexes have the potential to be developed as low-toxicity agents for lung cancer treatment.

## 1. Introduction

As a new class of nonplatinum metal complexes, ruthenium-based compounds possess valuable photophysical and photochemical properties and high structural diversity, which provide more direction for designing new ruthenium anticancer drugs [1, 2]. More importantly, ruthenium-based complexes often show better tumor cell selectivity than platinum metal complexes, in addition to low toxicity to normal cells and multiple anticancer mechanisms, thus making them attractive chemotherapeutic agents [3, 4]. So far, there are four ruthenium complexes with different ligands, that is, [ImH][trans-RuCl_4_(DMSO)(Im)] (NAMI-A) [5, 6], [IndH][trans-RuCl_4_(Ind)_2_] (KP1019) [7], KP133910 [8], and the Ru(II)-based photosensitizer, TLD1433 [9, 10], have progressed to different stages in clinical trials. In the review paper, Chen et al. pointed out that the selection of ligands plays a key role in the antitumor cell selectivity, targeting, antitumor activity, and mechanism of ruthenium compounds [3].

However, while there is a large number of studies mainly focused on Ru(II) complexes containing N, N- and C, N-chelated ligands, there is little research on the biological activities of O, O-chelating Ru(II) complexes. de Carvalho et al. found that ruthenium complex with xanthoxylin (RCX) ligand exhibited a potent cytotoxic effect in a panel of cancer cell lines in monolayer cultures and induced S-phase arrest and caused ERK1/2-mediated apoptosis in HepG2 cells through a p53-independent pathway [11]. Habtemariam et al. reported that the water-soluble Ru(II) acetylacetonate coordination compound ([Ru(acac)(pdto)]Cl, where pdto = 2,2′-[1,2-ethanediylbis-(sulfanediyl-2,1-ethanediyl)]dipyridine) showed a remarkable anti-E. histolytica activity *in vitro* with IC_50_ values of 60 nM [12]. Toledano-Magaña et al. reported a series of neutral Ru(II) complexes containing anionic O, O-chelating ligands (acetylacetonate derivatives), and their studies suggested some of these compounds possessed reasonable activity towards A2780 cells [13]. Furthermore, Bezerra and his partners synthesized some piplartine-containing ruthenium complexes and investigated the apoptosis inducing effect in human colon carcinoma HCT116 cells and underlying mechanisms by these ruthenium complexes-induce cell death [14, 15]. In our recent studies, we have synthesized three O, O-chelated ligand-bearing Ru(II) complexes, which are [Ru(bpy)_2_(SA)] (SA = salicylate), [Ru(phen)_2_(SA)] and [Ru(dmb)_2_(SA)], and found that these complexes induced apoptosis in A549 cells by targeting TrxR [16].

Notably, salicylic acid (SA) or o-hydroxybenzoic acid, a precursor of aspirin (AS), has been applied as a nonsteroidal anti-inflammatory drug (NSAID) [17]. Salicylate ligand has two hard and strong alkaline donor centers, which can promote chelation or metal bridging of medium to large cations [18]. As multifunctional ligands, SA and AS are attracting ever more attention for the treatment of cancer. For instance, the research of Wu et al. demonstrated that conjugation of SA or AS to the Ir(III) moiety could improve the cellular uptake efficacies of the Ir(III) complexes and achieve a high synergistic effect [19]. Liu and Dhar et al. reported that a novel Pt(IV) prodrug of cisplatin, asplatin, with the ligation of aspirin (*c*,*c*,*t*-[PtCl_2_(NH_3_)_2_(OH) (aspirin)]), demonstrated significantly higher cytotoxicity than cisplatin towards tumor cells and nearly completely overcame the drug resistance of cisplatin-resistant cells [20–22]. Recently, Kowalski reported a new rhenium compound with AS as the ligand, *fac*-[Re(CO)_3_(phen)(aspirin)], which exhibited activity against HeLa human cancer cells, and this anticancer activity could be linked to ROS production, and cell cycle disturbance followed by triggering an apoptotic pathway of cell death [23]. Similar exciting antitumor effect has also been found in other metal-based complexes of SA. For example, silver(I) [24–26] and copper(II) [18, 27] complexes with SA as the ligand exhibited strong antiproliferative activity against cancer cell lines.

**Figure 1:**
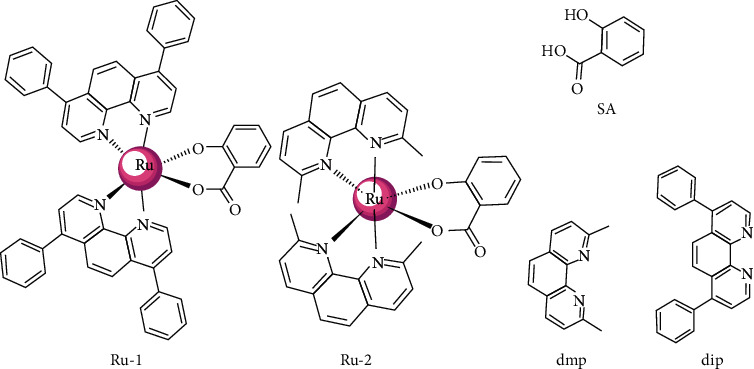
Structures of the Ru(II) complexes Ru(dip)_2_(SA) (**Ru-1**) and Ru(dmp)_2_(SA) (**Ru-2**) and the ligands of SA, dip, and dmp.

For enhancing anticancer effect, we sought to develop novel Ru(II) complexes containing O, O-chelated ligands and explored their biological functions. It is well-established that even small changes in the geometric structures of metal-based complexes could dramatically affect their biological activities, such as cyclometalated Ru(II) complexes and noncyclometalated Ru(II) complexes [28, 29]. Following this logic, we engineered two neutral O, O-chelated Ru(II) complexes, that is, [Ru(dip)_2_(SA)] (dip = 4,7-diphenyl-1,10-phenanthroline, **Ru-1**) and [Ru(dmp)_2_(SA)] (dmp = 2,9-dimethyl-1,10-phenanthroline, **Ru-2**) (Figure 1). The new synthesized Ru(II) complexes have been investigated for their solution stability, hydrophobic property, cellular uptake, and anticancer activity. Furthermore, to facilitate the next clinical translation, the *in vivo* toxicity of complex **Ru-1** was assessed using zebrafish embryo models.

## 2. Materials and Methods

### 2.1. Materials

Ultrapure MilliQ water was used in all experiments. DMSO, MTT, PBS, JC-1, DCFH-DA, PI, Hoechst 33342, Annexin V-FITC Apoptosis Detection Kit, QuantiPro™ BCA Assay Kit, ECL™ Start Western Blotting Detection Reagent, and endocytosis inhibitors including NaN_3_, DOG, Sucrose and Nystatin were purchased from Sigma-Aldrich (St. Louis, MO, USA). A549 (human lung carcinoma), HepG2 (human hepatocellular carcinoma), MCF-7 (breast cancer), HeLa (human cervical cancer), and BEAS-2B (immortalized human bronchial epithelial cells) cells were purchased from American Type Culture Collection (ATCC, Manassas, VA). Cisplatin was purchased from Acros. Ruthenium standard solution was purchased from Aladdin Chemistry Co. (Shanghai, China). Cell Mitochondria Isolation Kit was purchased from Beyotime (Shanghai, China). Antibodies were purchased from Cell Signaling Technology Company. Comet assay reagent kit was purchased from Trevigen (Gaithersburg, MD, USA).

### 2.2. Apparatus

Microanalyses were carried out with a Perkin-Elmer 240Q elemental analyzer. Electrospray ionization mass spectrometry (ESI-MS) were recorded on Agilent LC-MS6430B Spectrometer. Fourier Transform infrared (FTIR) spectra were recorded on a Shimadzu Fourier Transform Infrared Spectrophotometer IRT racer-100 in the 4000–400 cm^−1^ region. ^1^H NMR spectra were run on a Bruker AVANCE 400 spectrometer (400 MHz). UV-Vis spectra were recorded on a Perkin–Elmer Lambda-850 spectrophotometer (PerkinElmer, USA). Inductively Coupled Plasma Mass Spectrometry (ICP-MS) was performed by NEXION-300X (PerkinElmer, USA). Flow cytometry was performed by an EPICS XL-MCL (BECKMAN COULTER, USA). Fluorescence microscopy observation was performed by Ti-E (Nikon, Japan). Microplate was read by Tecan Infinite M200 pro. Protein bands were visualized using ChemiDocTM XRS Imaging System (Bio-Rad, USA). Statistical analysis was performed using SPSS statistical software, version 17 (SPSS Inc., Chicago, IL).

### 2.3. Synthesis of Complex [Ru(dip)_2_(SA)] (**Ru-1**)

Complex **Ru-1** was synthesized by reference to the procedure of complex [Ru(phen)_2_(SA)] according to our previous report [16], with *cis*-[Ru(dip)_2_Cl_2_] in place of *cis*-[Ru(phen)_2_Cl_2_]. Firstly, a mixture of *cis*-[Ru(dip)_2_Cl_2_]·2H_2_O (MW = 938.0, 0.057 g, 0.10 mmol), o-hydroxybenzoic acid (MW = 138.12, 0.0205 g, 0.15 mmol), and sodium hydroxide (MW = 40.0, 0.012 g, 0.30 mmol) were dissolved in ethanol and water mixed solution (5 mL, 5 mL), and then, the mixture at 78°C for 11 h under the protection of argon was reflowed and a clear red solution appeared. Secondly, the solution was cooled to room temperature and concentrated by rotary evaporator at 30°C. Finally, the precipitate was dried in vacuum and purified by chromatography over alumina (200 mesh) using ethanol/water (1 : 1, v/v) as an eluent, and complex **Ru-1** was obtained. Yield: 81.8%. ^1^H NMR (400 MHz, C_2_H_5_OH-*d*_6_) *δ* 9.71 (d, *J* = 5.5 Hz, 1H), 9.57 (d, *J* = 5.4 Hz, 1H), 8.20 (d, *J* = 8.0 Hz, 2H), 8.16–8.12 (m, 2H), 8.11–8.06 (m, 3H), 7.99 (d, *J* = 5.5 Hz, 1H), 7.94–7.87 (m, 2H), 7.75 (m, 4H), 7.66 (m, 4H), 7.62–7.46 (m, 11H), 7.39 (t, *J* = 5.2 Hz, 2H), 6.95 (ddd, *J* = 8.6, 6.8, 2.0 Hz, 1H), 6.67 (dd, *J* = 8.4 Hz, 1.3Hz, 1H), 6.41 (ddd, *J* = 8.0, 6.7, 1.3 Hz, 1H) ESI–MS (MeCN): *m*/*z* = 903.10 ([M + H]^+^). UV-Vis (*λ*/nm, *ε*/M^−1^·cm^−1^) (CH_3_CH_2_OH): 287(21275), 585(6125). Anal. calc. for C_55_H_36_N_4_O_3_Ru: C, 73.24; H, 4.02; N, 6.21; found: C, 73.51; H, 4.03; N, 6.21. FTIR (KBr, cm^−1^): 1597(*v*_as_, CO_2_, s), 1462(C–N, s), 1350(*v*_s_, CO_2_, m), 702(Ru−O, s).

### 2.4. Synthesis of Complex [Ru(dmp)_2_(SA)] (**Ru-2**)

Complex **Ru-2** was synthesized similarly to the procedure of complex **Ru-1**, with *cis*-[Ru(dmp)_2_Cl_2_] in place of *cis*-[Ru(dip)_2_Cl_2_]. Yield: 76.5%. ^1^H NMR (400 MHz, Pyridine-*d*_5_) *δ* 8.55 (d, *J* = 7.8 Hz, 1H), 8.00 (d, *J* = 7.9 Hz, 2H), 7.97–7.89 (m, 3H), 7.85–7.74 (m, 2H), 7.76–7.67 (m, 2H), 7.28 (d, *J* = 8.1 Hz, 2H), 7.03 (d, *J* = 8.2 Hz, 1H), 6.76 (ddd, *J* = 8.6, 6.7, 2.1 Hz, 1H), 6.40 (ddd, *J* = 7.1, 6.7, 1.2 Hz, 1H), 6.14 (dd, *J* = 8.4, 1.3 Hz, 1H), 3.02 (s, 3H), 2.97 (s, 3H), 1.95 (s, 3H), 1.94 (s, 3H). ESI-MS (MeCN): *m*/*z* = 655.09 ([M + H]^+^). UV-Vis (*λ*/nm, *ε*/M^−1^·cm^−1^) (CH_3_CH_2_OH): 273(38250), 554(6500). Anal. calc. for C_35_H_28_N_4_O_3_Ru: C, 64.31; H, 4.32; N,8.57; found: C, 64.29; H, 4.31; N, 8.60. FTIR (KBr, cm^−1^): 1597(as, CO_2_, s), 1462(C–N, s), 1350(s, CO_2_, m), 702(Ru−O, s).

### 2.5. Cell Culture Conditions and MTT Assay

All cell lines were cultured in Roswell Park Memorial Institute 1640 culture media supplemented with 10% fetal bovine serum and incubated at 37°C in a 5% CO_2_ incubator. The IC_50_ values in Table 1 were measured by MTT assay according to our previous report [16].

### 2.6. Cellular Uptake and Nuclear Localization

A549 cells were seeded into six-well plates (5.0 × 10^5^ cells per well) and grown overnight at 37°C in a 5% CO_2_ incubator. The cells were incubated with the different concentrations (5, 10, 15, and 20 *μ*M) of Ru(II) complexes for different time intervals (1, 3, and 6 h). After the incubation, the cells were harvested and washed twice with PBS. Cell Mitochondria Isolation Kit was used to extract the nuclear, mitochondrial, and cytoplasmic fractions of the A549 cells. The pellets were digested with 3 mL concentrated nitric acid and 1 mL perhydrol for 24 h and then diluted to 5 mL with ultrapure water. Finally, ICP-MS was used to determine the amount of Ru(II) complexes uptaken by A549 cells.

### 2.7. Lipophilicity Measurements

Log *P*_o/w_ is the partition coefficient between octanol and water. Briefly, a suitable amount of stock solution of the Ru(II) complex in aqueous NaCl was added to an equal volume of octanol, and the mixture was shaken for 48 h at 200 rpm at 25°C to allow partitioning. The aqueous layer was separated from the octanol layer after the sample was centrifuged at 3000 rpm for 10 min. The Ru(II) content in the aqueous layer was measured by ICP-MS. Finally, Log *P*_o/w_ values were calculated according to the equation of Log *P*_o/w_ = Log ([Ru]_o_/[Ru]_w_).

### 2.8. Apoptosis Assay

First, A549 cells were incubated in six-well plates for 12 h and then exposed to Ru(II) complexes for 24 h. After incubation, cell nuclei were stained with Hoechst 33342 (5 *μ*g/mL) for 10 min, washed twice with PBS, and then photographed using an inverted fluorescence microscope. Second, after incubation with different concentrations (5, 10, and 20 *μ*M) of **Ru-1** and **Ru-2** for 24 h, respectively, cells were harvested and washed twice with PBS and then resuspended in 500 *μ*L binding buffer. The suspension was stained with 5 *μ*L Annexin V-FITC and 10 *μ*L PI at room temperature for 15 min and then analyzed using the flow cytometer.

### 2.9. MMP and ROS Determination

The mitochondrial membrane potential (MMP) of A549 cells was analyzed by using an inverted fluorescence microscope and flow cytometry. After pretreatment with different concentrations (5, 10, and 20 *μ*M) of **Ru-1** and **Ru-2** for 12 h, the A549 cells were trypsinized and washed twice with PBS. For microscope observation, the collected cells were incubated in complete medium containing JC-1 (10 *μ*g/mL) for 30 min and washed with PBS twice and then imaged by an inverted fluorescence microscope (Nikon, Japan). For flow cytometry analysis, the cells were trypsinized and washed twice with PBS and then incubated in 500 *μ*L PBS containing JC-1 (10 *μ*g/mL) for 30 min at 37°C. The cells were analyzed by flow cytometer immediately. The intracellular ROS level of A549 cells was detected after DCFH-DA stain, as illustrated in our new publication [16, 30].

### 2.10. Comet Assay and Western Blotting Assay

Single-cell gel electrophoresis was performed to detect DNA damage, as previously described [29]. Comet assay was performed according to the manufacturer's instructions. DNA was stained with SYBR Green I (Trevigen) and imaged under an inverted fluorescence microscope.

The effects of **Ru-1** on the expression levels of proteins associated with caspase, Bcl-2 family proteins, and DNA damage relative proteins were examined by western blotting assay according to our previously reported method [31]. Glyceraldehyde-3-phosphate dehydrogenase (GAPDH) was used as an internal control. The protein concentrations were determined by BCA Protein Assay Kit.

### 2.11. *In Vivo* Toxicity Assay

The in *vivo* toxicity of complex **Ru-1** was assessed on developing zebrafish embryos. Zebrafish embryos were provided by the Zebrafish Platform of Affiliated hospital of Guangdong Medical University. Zebrafish embryos were incubated in 12-well plates with 2 mL solutions containing various doses of complex **Ru-1** (0, 12.5, 25, 50, 100, and 200 *μ*M) in water. An inverted microscope was used to observe the hatching and growth of the zebrafish embryos with and without **Ru-1** every 24 h. The ethical protocols used for the *in vivo* zebrafish embryo study were performed in line with the ethics regulations of Guangdong Medical University.

## 3. Results and Discussion

### 3.1. Synthesis, Characterization, and Solution Stability

Complexes **Ru-1** and **Ru-2** were synthesized by the reaction of cis-[Ru(N-N)_2_Cl_2_] (N-N = dip, dmp) and salicylic acid in the presence of sodium hydroxide. The crude product was purified by chromatography over alumina. The obtained compounds were analyzed by elemental analysis, ESI-MS, ^1^H NMR, FTIR, and UV-Vis absorption spectroscopy, and the results were shown in Figures S1–S7 in Supplementary Materials. The stability of **Ru-1** and **Ru-2** in water and CH_3_CN solution at 298 K was analyzed by UV-Vis absorption spectroscopy (Figures S3–S5). As demonstrated in Figures S4 and S5, there is no significant change in the UV-Vis absorption spectra of **Ru-1** and **Ru-2** at 298 K over 24 h, indicating that **Ru-1** and **Ru-2** are sufficiently stable for the majority of clinical applications. In the UV-visible spectral regions, both complexes consist of two well-resolved absorption bands. In the visible region, these two complexes present a strong MLCT transition at 500–600 nm attributed to the overlap Ru(d*π*)-dip or dmp (*π*^*∗*^) and Ru(d*π*)-SA (*π*^*∗*^). The intense band occurring between 250 nm and 300 nm is attributed to an intra-ligand (IL) *π*-*π*^*∗*^ transition by comparing with the spectra of other Ru(II)-polypyridyl complexes [32–34].

The obtained FTIR spectra of **Ru-1** and **Ru-2** in KBr pellets are shown in Figures S6 and S7. In the FTIR spectrum of **Ru-1** and **Ru-2**, the absorptions due to the stretching vibrations of the carboxylato unit have been recognized at 1597 and 1350 cm^−1^ [35, 36]. The band at 702 cm^−1^ is attributed to the Ru–O stretch of **Ru-1**. Additionally, the appearance of the peak at 559 cm^−1^ corresponding to the Ru–O stretch of **Ru-2** is consistent with Ru(II) coordination [37]. The bands at ca. 1462 cm^−1^ and 1465 cm^−1^ are the stretching vibrations of the C–N group of the ligand dip of **Ru-1** and dmp of **Ru-2**, separately [38, 39]. In addition, peak observed at 1365 cm^−1^ is attributed to the methyl of dmp of **Ru-2** [38], which distinguished the IR spectrum of **Ru-2** from that of **Ru-1**.

Investigating the hydrolysis in the presence of biorelevant molecules is very important to assess the chemical stability and biological activities of these title complexes. Hence, the stability of complexes **Ru-1** and **Ru-2** towards bovine serum albumin (BSA) and glutathione (GSH) was also determined. As shown in Figures S8 and S9, after 12 h of incubation with excessive GSH or BSA, respectively, there was no significant change observed in the characteristic absorption of **Ru-1** and **Ru-2**, which suggested that **Ru-1** and **Ru-2** are quite stable in PBS buffer. Also, it implied that **Ru-1** and **Ru-2** were inactive towards GSH and BSA.

### 3.2. *In Vitro* Cytotoxicity Assay

To evaluate the *in vitro* anticancer activities of **Ru-1** and **Ru-2**, the ligand SA and two synthetic precursors *cis*-[Ru(dip)_2_Cl_2_] and *cis*-[Ru(dmp)_2_Cl_2_] were incubated with four cancer cell lines, A549, HepG2, MCF-7, HeLa, and one normal cell line BEAS-2B, at various concentrations for 48 h. The cell viability was obtained by the MTT assay. The measured IC_50_ values were listed in Table 1. As shown, while the ligand SA, *cis*-[Ru(dip)_2_Cl_2_] and *cis*-[Ru(dmp)_2_Cl_2_] displayed no/low cytotoxicity, **Ru-1** and **Ru-2** recorded anticancer effect in all of the tested cell lines, suggesting that the coordination of polypyridine-Ru(II) center with SA is a crucial factor for the observed cytotoxic activities. It should be noted that although **Ru-1** (IC_50_ = 54.3 ± 3.4 *μ*M) and **Ru-2** (IC_50_ = 48.1 ± 3.7 *μ*M) show comparable low cytotoxicity towards normal BEAS-2B cells, **Ru-1** displays higher anticancer activity than **Ru-2**, especially for A549 cell line. Hence, **Ru-1** displays better selectivity between cancer cells and normal cells, indicating that it has a preferable therapeutic profile against cancer, especially lung cancer cells. This is further supported by the selectivity index (SI) assay (Table 1). Compared with **Ru-2** (1.6) and cisplatin (0.8), **Ru-1** (4.9) demonstrates significantly higher safety profile.

### 3.3. Cellular Uptake and Localization

**Figure 2:**
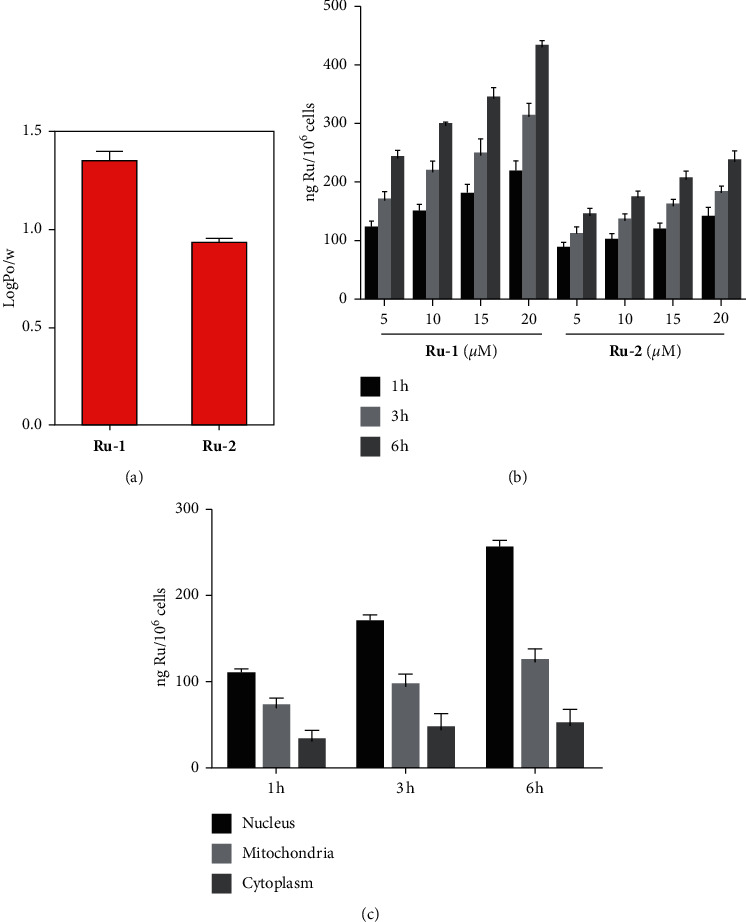
(a) The logP_o/w_ values of **Ru-1** and **Ru-2**. (b) Cellular ruthenium concentrations determined in A549 cells after 1, 3, and 6 h of incubation with **Ru-1** and **Ru-2** at 5, 10, 15, and 20 *μ*M, respectively. (c) Subcellular distribution of **Ru-1** in A549 cells after incubation with 20 *μ*M of **Ru-1** for different times.

The lipophilicity of a drug plays a vital role in its cytotoxicity [29, 40–42]. According to our studies, the cellular uptake and cytotoxicity of a ruthenium complex is positively correlated with its lipophilicity [29, 30]. Log *P*_o/w_ values were used to estimate the lipophilicity quantitatively. As illustrated in Figure 2(a), complex **Ru-1** (log *P*_o/w_ = 1.4 ± 0.1) shows higher log *P*_o/w_ value than that of **Ru-2** (log *P*_o/w_ = 0.9 ± 0.02), suggesting **Ru-1** has higher lipophilicity. The above result is in line with the report that there is a positive correlation between hydrophobicity and cytotoxicity for metal-based anticancer agents [42–44].

It is generally acknowledged that the cellular uptake feature of transition metal-based compounds is an essential factor for their biological activities [45–47]. As repeatedly reported by previous ruthenium complex-related studies, the more cellular uptake results in higher cytotoxicity [29, 30, 48]. The cellular ruthenium concentration was determined by using ICP-MS after 1, 3, and 6 h of exposure to 5, 10, 15, and 20 *μ*M Ru(II) complexes (Figure 2), respectively, and the results were reported as ng of ruthenium per 10^6^ cell number. As expected, **Ru-1** exhibits higher cellular uptake than that of **Ru-2** during all the tested time points and drug concentrations (Figure 2(b) and Table S1). In a short time (1 h), the uptake levels were relatively low for **Ru-1** (120.3 ± 9.5 ng) and **Ru-2** (85.4 ± 9.3 ng) at low drug concentration (5 *μ*M). With the extension of time and increase of concentration, the uptake levels for both **Ru-1** and **Ru-2** increased. The maximum cellular uptake was reached after 6 h of exposure to **Ru-1** (432.5 ± 12.4 ng) and **Ru-2** (235.2 ± 16.6 ng) at the concentration of 20 *μ*M, and **Ru-1** has a 1.8-fold better cell entry than **Ru-2**. The subcellular distribution of **Ru-1** in A549 cells was further studied. As displayed in Figure 2(c), **Ru-1** was mostly accumulated in the cell nucleus, followed by mitochondria. These results imply that **Ru-1** may lead to mitochondria and nucleus damage and subsequent cancer cell apoptosis.

### 3.4. Complexes **Ru-1** and **Ru-2** Induced A549 Cell Apoptosis

**Figure 3:**
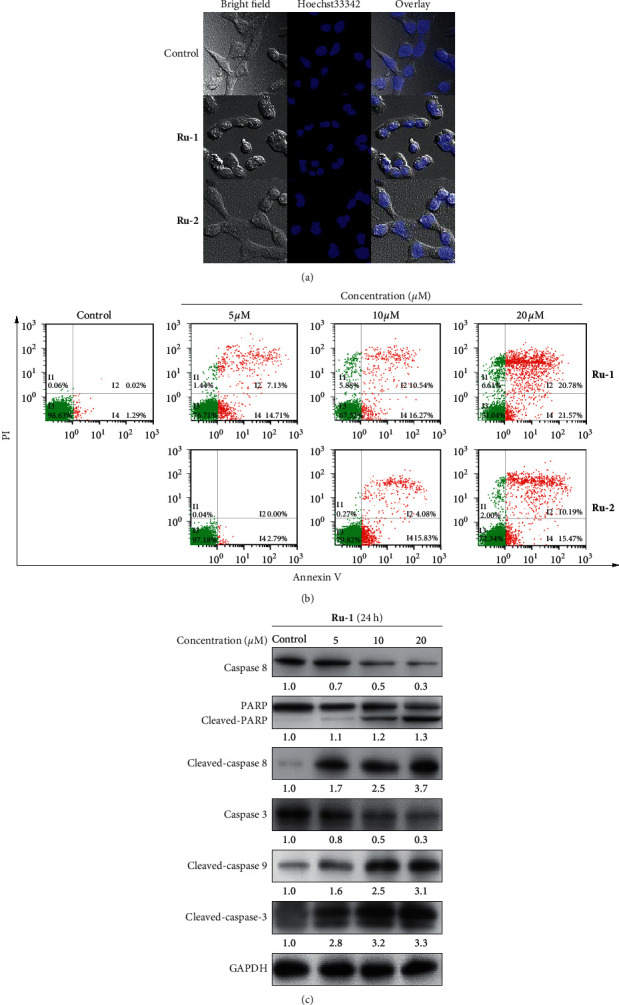
(a) A549 cells stained with Hoechst 33342 after treatment of Ru(II) complexes at 20 *μΜ* for 24 h. (b) Apoptosis in A549 cells was detected by Annexin V/PI assay after coincubation with different concentrations of Ru(II) complexes for 24 h (I2: late apoptotic or necrotic cells, I3: living cells, and I4: early apoptotic cells). (c) The expression levels of caspase-3/8, PARP, cleaved caspase-3/8/9, and PARP were evaluated in a concentration-dependent manner with **Ru-1** treatment for 24 h.

Apoptosis, an active form of cell death, plays a vital role in cell development and survival by clearing away damaged or unwanted cells [49]. Apoptosis is an approach by which many chemical agents exert anti-cancer effect [38, 50]. Based on the apparent proliferation inhibition of **Ru-1** and **Ru-2** on A549 cells, their impact on apoptosis induction was further studied via Hoechst 33342 staining technique. As presented in Figure 3(a), after treatment with both **Ru-1** and **Ru-2**, apoptotic features including chromatin condensation, nuclear fragmentation, and plasma membrane blebbing were observed. Also, at the same concentration, complex **Ru-1** induced more severe apoptosis than **Ru-2** in the tested cells. To further ascertain the apoptosis induced by these Ru(II) complexes, flow cytometric analysis was carried out. As shown in Figure 3(b), both **Ru-1** and **Ru-2** significantly induced apoptosis in A549 cells in a concentration-dependent manner. After treatment with **Ru-1** and **Ru-2** at 5, 10, and 20 *μ*M for 24 h, respectively, the percentages of late apoptotic/necrotic cells increased from 7.13% (5 *μ*M) to 20.78% (20 *μ*M) for **Ru-1** and from 0.00% (5 *μ*M) to 10.19% (20 *μ*M) for **Ru-2**, and the percentages of early apoptotic A549 cells were 14.71% (5 *μ*M), 16.27% (10 *μ*M), and 21.57% (20 *μ*M) for **Ru-1** and 2.79% (5 *μ*M), 15.83% (10 *μ*M), and 15.47% (20 *μ*M) for **Ru-2**, respectively.

When apoptosis occurred, the expression of apoptosis-related proteins might change. So, the effect of **Ru-1** on key apoptosis protein caspase-3/8/9 and poly (ADP-ribose) polymerase (PARP) were analyzed by western blotting (Figure 3(c)). As demonstrated in Figure 3(c), after treatment with **Ru-1** for 24 h, the expression of cleaved-PARP, cleaved caspase-3/8/9 increased, and the expression of total caspase-3/8 decreased in a concentration-dependent manner, which illustrated that both intrinsic and extrinsic apoptotic pathways were involved in **Ru-1**-induced apoptosis of A549 cells. In conclusion, complexes **Ru-1** and **Ru-2** induced apoptosis in A549 cells probably through caspase-dependent extrinsic and intrinsic pathway.

### 3.5. Complexes **Ru-1** and **Ru-2** Induced the Decrease of MMP

**Figure 4:**
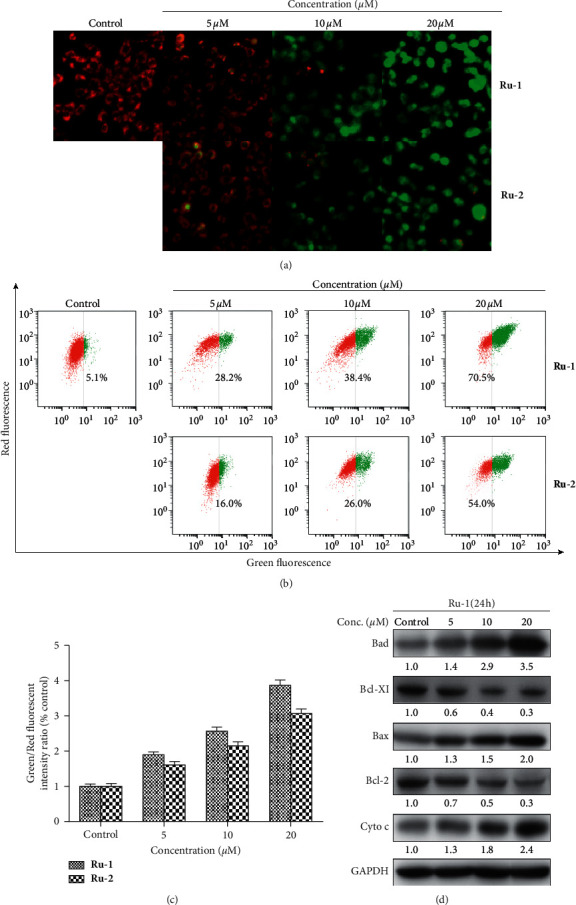
(a) Fluorescence microscopy analysis of cellular MMP level by JC-1 staining after Ru(II) complexes treatment for 12 h. (b) Flow cytometry analysis of cellular MMP level after Ru(II) complexes treatment for 12 h. (c) The quantification of green/red fluorescent intensity ratio by JC-1 staining after 5, 10, and 20 *μ*M of **Ru-1** and **Ru-2** treatment for 12 h. (d) The effect of **Ru-1** on the expression of Bcl-2 family proteins and cytochrome c in the cytosol.

Mitochondrion, which controls the energy production in most eukaryotic cells, is one of the major cell signaling center and plays a vital role in various cellular activities [51, 52]. Mitochondrial dysfunction, such as the decline of mitochondrial membrane potential (MMP), is an important indicator for apoptosis detection [53]. To test the effect of **Ru-1** and **Ru-2** on MMP, fluorescent dye JC-1-based MMP evaluation was employed. As Figure 4(a) illustrated, comparing with the untreated cells, after incubation with 5, 10, and 20 *μ*M of **Ru-1** or **Ru-2** for 12 h, the A549 cells presented a significant red-to-green color shift in a concentration-dependent manner, suggesting the decline of MMP. Meanwhile, flow cytometry was employed to provide further information about the quantificational changes of MMP. As shown in Figure 4(b), after incubation with 5, 10, and 20 *μ*M of **Ru-1** or **Ru-2**, the percentage of green fluorescence underwent a dramatic increase from 5.1% to 70.5% for **Ru-1** and from 5.1% to 54.0% for **Ru-2**, respectively. The quantification of green/red fluorescent intensity ratio by JC-1 staining after 5, 10, and 20 *μ*M of **Ru-1** and **Ru-2** treatment for 12 h is shown in Figure 4(c).

To investigate whether the mitochondrial pathways involved in **Ru-1**-induced apoptosis, western blotting was further performed. As presented in Figure 4(d), after coincubation with **Ru-1**, while the proapoptotic protein Bax and Bad displayed increased expression, the expression levels of the antiapoptotic proteins Bcl-2 and Bcl-xl were both downregulated. The reduction of the ratios of Bcl-2/Bax and Bcl-xl/Bad results in the release of cytochrome c into the cytosol [54, 55]. The apparent enhancement of cytochrome c expression in cytosol can be observed in Figure 4(d). The above results confirmed that **Ru-1** and **Ru-2** could induce the reduction of MMP, and the mitochondrial pathway was involved in **Ru-1**-induced apoptosis in A549 cells.

### 3.6. Complexes **Ru-1** and **Ru-2** Activated Intracellular ROS Generation

**Figure 5:**
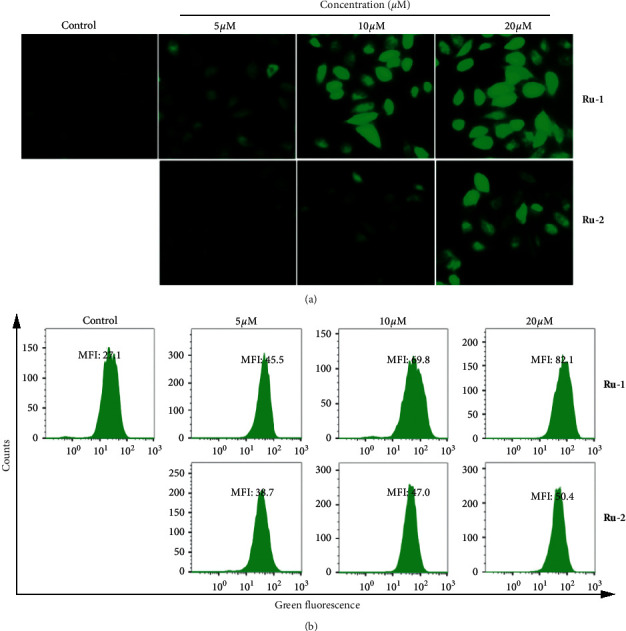
(a) The intracellular ROS level regulated by **Ru-1** and **Ru-2** was detected by an inverted fluorescence microscope. (b) Flow cytometry analysis of intracellular ROS level by DCFH-DA staining after different concentrations of Ru(II) complexes treatment for 12 h.

Increasing intracellular ROS levels can trigger cell apoptosis [56]. Various Ru(II) complexes have been reported being able to enhance the intracellular ROS levels markedly [29, 30]. To explore the roles of ROS in Ru(II) complex-induced cell apoptosis, we detected intracellular ROS levels by using an inverted fluorescence microscope and flow cytometry after staining A549 cells with DCFH-DA fluorescent dye. The results in Figure 5(a) displayed that, for both **Ru-1** and **Ru-2** groups, with the increase in the treatment concentration, a significant enhancement of DCF fluorescence signals could be observed after 12 h incubation. These results indicated that **Ru-1** and **Ru-2** induced overgeneration of ROS in A549 cells in a concentration-dependent manner. The quantitative result in Figure 5(b) exhibited that, at a concentration of 20 *μ*M, coincubation with both **Ru-1** and **Ru-2** for 12 h could result in significant enhancement of the mean fluorescent intensity (MFI), with about 3.0- and 1.9-fold higher than the control group for **Ru-1** and **Ru-2**, respectively.

As reported, mitochondria are both a source and target of ROS [57]. The complexes studied in this work can induce the decline of mitochondrial membrane potential (MMP) and results in mitochondrial dysfunction, which causes the damage of respiratory chain, and generates radical and nonradical species such as superoxide anion (O^2−^) and hydrogen peroxide (H_2_O_2_). Based on the above information, these Ru(II) complexes may stimulate the mitochondria-based ROS generation. This speculation correlates with our previous publication [31], and the Ru(II) complex-triggered ROS generation could be effectively blocked by using cyclosporine A (CsA), a confirmed mitochondrial permeability transition pore (MPTP) opening inhibitor.

### 3.7. Complexes **Ru-1** and **Ru-2** Triggered DNA Damage

**Figure 6:**
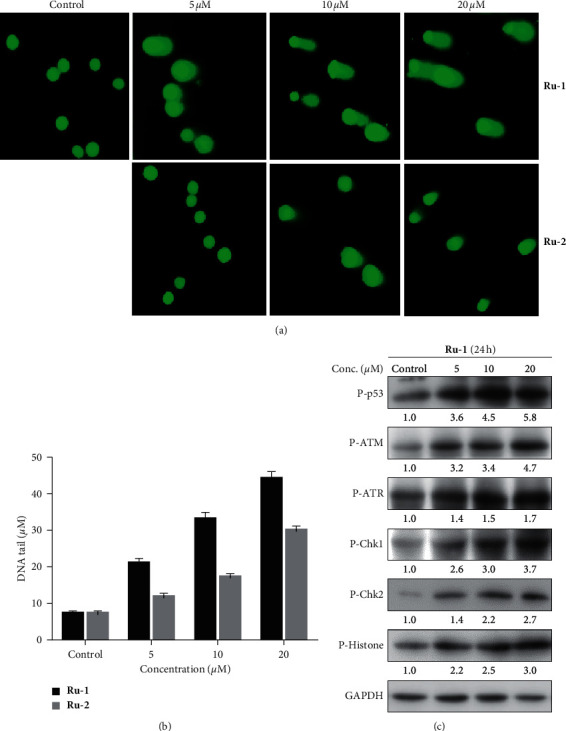
(a) DNA fragmentation triggered by **Ru-1** and **Ru-2** was determined by comet assay. (b) Quantification of DNA tails in the comet assay. The length of DNA tails in microscopy images was quantified by ImageJ. (c) The expression levels of phosphorylated proteins p53, ATM/ATR, Chk1/Chk2, and histone were determined by western blotting.

Excessive intracellular ROS can activate DNA damaged sensor proteins [58] and lead to DNA damage, an essential hallmark of cell apoptosis [59]. Indeed, the DNA damage effect has been observed in many previously developed Ru(II) complexes [29, 30]. To determine whether or not these complexes can induce DNA damage, the single-cell gel electrophoresis assay (comet assay) was conducted in our study. Comet assay is proved to be a rapid, simple, convenient, and straightforward method for assessing DNA damage in single cells, and the length of the comet tail represents the level of DNA damage. Also, the comet assay (DNA fragments appearance) could serve as a mark for apoptosis assessment. As shown in Figure 6(a), in the control group, there was no comet-like cell observed. After A549 cells were incubated with **Ru-1** (10 *μ*M) and **Ru-2** (10 *μ*M) for 12 h, the cells presented well-formed comet tails, demonstrating the existence of severe DNA fragmentation. Increasing the concentration to 20 *μ*M recorded markedly higher levels of comet tails, showing that more DNA fragmentation has occurred. For quantitative comparison, the length of DNA tails in microscopy images was quantified by ImageJ, and the quantification of DNA tails in the comet assay was displayed in Figure 6(b). As expected, complex **Ru-1** induced more DNA damage than that induced by **Ru-2** based on the same external concentrations (Figure 6(b)).

In addition, **Ru-1**-induced DNA damage was further confirmed by analyzing common DNA damage markers via western blotting assay (Figure 6(c)), as evidenced by the upregulation of the phosphorylation levels of ATM, ATR, histone, and p53 in a concentration-dependent manner. Meanwhile, Chk1 and Chk2 were activated, accompanied by the increased phosphorylation levels of Chk1 and Chk2. The induction of p53 in response to DNA damage is synergistic with ATM/ATR, which can recruit Chk1/Chk2 and subsequently activate downstream cell cycle arrest-associated effector CDC25, causing cell apoptosis [60–62]. Therefore, these results demonstrated that **Ru-1** and **Ru-2** can trigger DNA fragmentation, that is, the apoptosis-mediated cell death.

The comet tail observed does not mean that the ruthenium complexes directly damage DNA. As reported, the excess intracellular ROS could attack DNA, resulting in DNA damage [58, 59]. So, the observed DNA damage may result from the increased intracellular ROS levels. This has been confirmed by our previous study (the cyclometalated Ru(II) *β*-carboline complexes could induce DNA damage through ROS overproduction) [29]. In addition, according to our previous studies [29, 63, 64], we speculate that the binding affinity of the title complexes towards DNA is not very strong because they do not have a large planar aromatic ligand (intercalative), such as dppz, dpq, and pip, so the interaction between them and DNA is not supposed to be the primary reason of apoptosis.

The apoptosis-mediated cell death has been commonly measured using the comet assay to detect DNA damage of cells after treatment with complexes. Comets with almost all DNA in the tail are often referred to as “hedgehog” comets and are widely assumed to represent apoptotic cells. In summary, the title complexes could induce mitochondrial dysfunction and the generation of intracellular ROS, which may indirectly lead to DNA damage.

### 3.8. Complex **Ru-1** Showed Little Toxicity to Zebrafish Embryos

**Figure 7:**
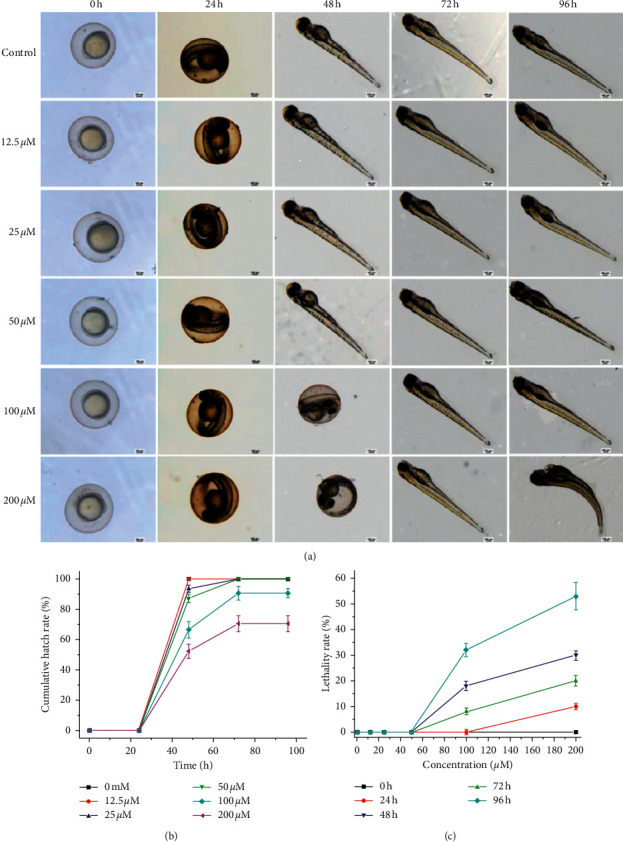
(a) Ecotoxicology of **Ru-1** to zebrafish embryo at various concentrations for 96 h on a 4× objective lens in the microscope. (b) Cumulative hatching rate of zebrafish embryos in the presence/absence of **Ru-1** at various concentrations every 24 h. (c) Lethality rate of zebrafish embryos in the presence/absence of **Ru-1** at different concentrations every 24 h.

Currently, the zebrafish model is attracting unprecedented interests in biomedical research due to its high reproductive rate, short growth period, and high homology with human DNA [65–67]. Thus, in this work, the developing zebrafish embryos were used to assess the in vivo toxicity of Ru(II) complex. As shown in Figures 7(b) and 7(c), when treated with **Ru-1** with the concentration close to IC_50_ value (12.5 *μ*M), the cumulative hatch rate and lethality rate display no difference compared with those of the control group. When the concentration of **Ru-1** was lower than 50 *μ*M, all zebrafish embryos can develop into juvenile zebrafish after 96 h. Even if the concentration of **Ru-1** was up to 100 *μ*M, the cumulative hatch rate was acceptable, and the lethality rate was lower than 35% after 96 h. Furthermore, when the concentration of **Ru-1** was lower than 100 *μ*M, no malformation was observed at 96 h (Figure 7(a)). However, when the concentration of **Ru-1** was up to 200 *μ*M, even after 72 h treatment, there were lower than 70% cumulative hatch and dramatically increased lethality rate (Figures 7(b) and 7(c)). What is more, unhealthy features such as pericardial cysts and spine curvature were notably observed at 96 h (Figure 7(a)). Therefore, the *in vivo* toxicity of **Ru-1** was concentration-dependent, and no apparent side effects were found with the treatment concentrations from 12.5 to 50 *μ*M. The above results indicated that complex **Ru-1** showed little toxicity to zebrafish embryos. Since hypotoxicity to normal cells or organs is essential for developing anticancer agents, complex **Ru-1** hold great potential to be developed as a low-toxicity agent against lung cancer cells.

## 4. Conclusions

In summary, our study extensively evaluated the anticancer effect of two novel Ru(II) complexes containing O, O-chelated ligands in A549 human lung cancer cells, which was mediated through inducing cell apoptosis. In addition, our results provided evidence that these complexes enhanced the level of intracellular ROS, induced a decrease of MMP, and indirectly led to DNA damage. Also, further studies showed that **Ru-1** activated the caspase family proteins and PARP, downregulated the levels of the antiapoptotic protein Bcl-2 and Bcl-xl, upregulated the levels of the proapoptotic proteins Bax and Bad, and induced the release of cytochrome *c* in A549 cells. More importantly, **Ru-1** exhibited low toxicity towards both normal BEAS-2B cells *in vitro* and zebrafish embryos *in vivo*. Altogether, complex **Ru-1** can induce cell apoptosis via the mitochondrial pathway, which involves mitochondrial dysfunction, ROS accumulation, and caspase-related family members' activation. This work, therefore, suggested that the title Ru(II) complexes have the potential to develop into lung cancer therapeutic agents with safety profiles.

## Figures and Tables

**Table 1:** Cytotoxic effects of ruthenium (II) complexes on human cancer and normal cell lines^a^.

Complexes	IC_50_ (*μ*M)					
	A549	HeLa	MCF-7	HepG2	BEAS-2B	SI
Ru(dip)_2_Cl_2_	138.4 ± 6.7	115.6 ± 6.8	134.9 ± 7.9	108.5 ± 5.4	—	—
Ru(dmp)_2_Cl_2_	>200	>200	>200	169.3 ± 6.2	—	—
SA	>200	>200	>200	>200	—	—
**Ru-1**	11.3 ± 1.1^b^	20.1 ± 1.3^b^	29.5 ± 2.2^b^	15.4 ± 1.0	54.3 ± 3.4^b^	4.9
**Ru-2**	30.1 ± 1.2^b^	32.5 ± 2.4^b^	45.7 ± 3.7^b^	26.4 ± 2.1	48.1 ± 3.7^b^	1.6
Cisplatin	27.2 ± 1.4	18.3 ± 1.2	16.2 ± 2.0	30.2 ± 2.0	21.4 ± 1.5	0.8

^a^Cells were treated with various concentrations of complexes for 48 h. SI (selectivity index) = IC_50_ (BEAS-2B)/IC_50_ (A549).^b^*p* < 0.01 represents significant differences compared with BEAS-2B.

## Data Availability

The data used to support the findings of this study are given in the supplementary information file.

## Supplementary Materials

Supplementary Materials Figures S1 and S2: ESI-MS spectra of **Ru-1** and **Ru-2** in MeCN. Figure S3: absorption spectra of **Ru-1** (20 *μ*M) and **Ru-2** (20 *μ*M) in CH_3_CN and H_2_O at 298 K for 24 h. Figure S4: absorption spectra of **Ru-1** (20 *μ*M) and **Ru-2** (20 *μ*M) in CH_3_CN at 298 K for 24 h. Figure S5: absorption spectra of **Ru-1** (20 *μ*M) and **Ru-2** (20 *μ*M) in H_2_O at 298 K for 24 h. Figures S6 and S7: FTIR spectra of **Ru-1** and **Ru-2** at a 1 : 150 dilution in KBr. Figures S8 and S9: UV/vis absorption of **Ru-1** and **Ru-2** in the absence and presence of GSH or BSA. Table S1: intracellular content of **Ru-1** or **Ru-2** were determined by ICP-MS at different times and concentration.
