# Supplementary material for: Ru(II) Complexes Bearing O, O-Chelated Ligands Induced Apoptosis in A549 Cells through the Mitochondrial Apoptotic Pathway
Source: Bioinorg Chem Appl. 2020 Aug 17;2020:8890950. doi: 10.1155/2020/8890950 (PMC7448123; doi:10.1155/2020/8890950)
Supplement: Supplementary Materials — Figures S1 and S2: ESI-MS spectra of Ru-1 and Ru-2 in MeCN. Figure S3: absorption spectra of Ru-1 (20 μM) and Ru-2 (20 μM) in CH3CN and H2O at 298 K for 24 h. Figure S4: absorption spectra of Ru-1 (20 μM) and Ru-2 (20 μM) in CH3CN at 298 K for 24 h. Figure S5: absorption spectra of Ru-1 (20 μM) and Ru-2 (20 μM) in H2O at 298 K for 24 h. Figures S6 and S7: FTIR spectra of Ru-1 and Ru-2 at a 1 : 150 dilution in KBr. Figures S8 and S9: UV/vis absorption of Ru-1 and Ru-2 in the absence and presence of GSH or BSA. Table S1: intracellular content of Ru-1 or Ru-2 were determined by ICP-MS at different times and concentration. [file 8890950.f1.docx]

**Supplementary Material**

**Ru(II) complexes bearing O, O-chelated ligands induced apoptosis in A549 cells through the mitochondrial apoptotic pathway**

**Jincan Chen^1^, Jie Wang^1^, Yuanyuan Deng^1^, Tao Wang^2^, Tifang Miao^3^, Chengpeng Li^4^, Xianhong Cai^1^, Ying Liu^1^, Justin Henri^5^ and Lanmei Chen ^1,*^**

^1^ Guangdong Key Laboratory for Research and Development of Nature Drugs, School of Pharmacy, Guangdong Medical University, Zhanjiang 524023, China

^2^ School of Nursing, Zhengzhou University, Zhengzhou, China, 450001

^3^ School of Chemistry and Materials Science, Huaibei Normal University, Huaibei 235000, China.

^4^ The Public Service Platform of South China Sea for R&D Marine Biomedicine Resources, Marine Biomedical Research Institute, Guangdong Medical University, Zhanjiang 524023, China

^5^ School of Medicine Deakin University, Geelong, Victoria 3128, Australia

**^*^** Correspondence: lanmeichen@126.com; Tel.: +86-759-2388568

**Figure S1.** ESI-MS spectra of **Ru-1** in MeCN, Calcd. for C_55_H_36_N_4_O_3_Ru 901.988 ([M]), found m/z = 903.105 ([M+H]^+^).

**Figure S2.** ESI-MS spectra of **Ru-2** in MeCN, Calcd. for C_35_H_28_N_4_O_3_Ru 653.704 ([M]), found m/z = 655.068 ([M+H]^+^), m/z = 1309.209 ([2M+H]^+^).

**Figure S3.** Absorption spectra of (A) **Ru-1** (20 μM) and (B) **Ru-2** (20 μM) in CH_3_CN and H_2_O at 298 K for 24 h.

**Figure S4.** Absorption spectra of (A) **Ru-1** (20 μM) and (B) **Ru-2** (20 μM) in CH_3_CN at 298 K for 24 h.

**Figure S5.** Absorption spectra of (A) **Ru-1** (20 μM) and (B) **Ru-2** (20 μM) in H_2_O at 298 K for 24 h.

**Figure S6**. FTIR spectra of **Ru-1** at a 1:150 dilution in KBr.

**Figure S7.** FTIR spectra of **Ru-2** at a 1:150 dilution in KBr.


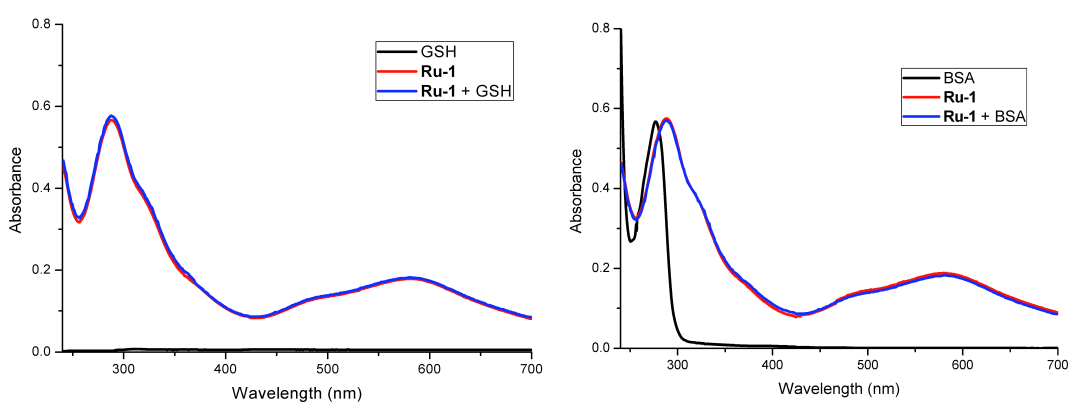


**Figure S8.** UV/vis absorption of **Ru-1** in the absence and presence of (A) GSH or (B) BSA. All spectra were acquired 12 h after GSH or BSA addition at 37 °C in PBS buffer (pH 7.4).


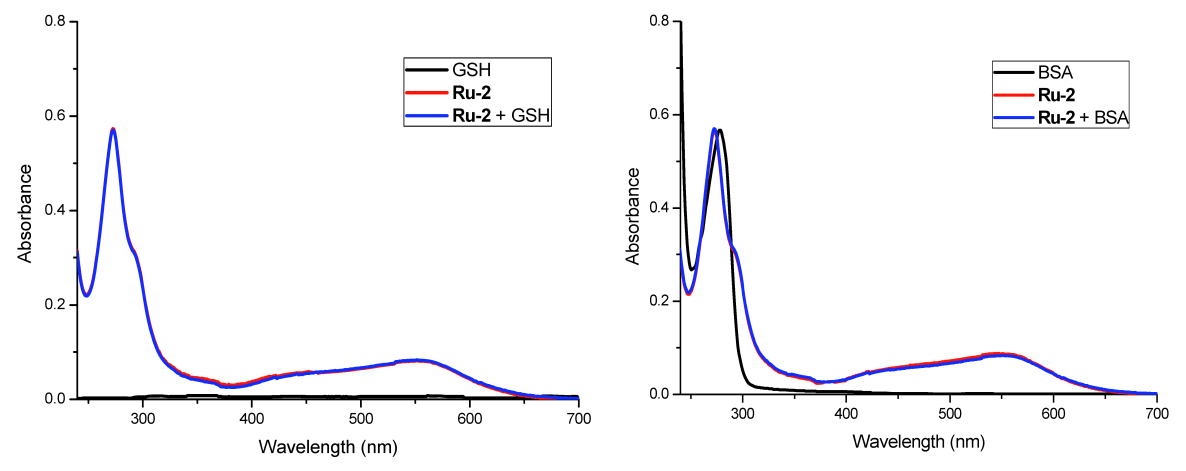


**Figure S9.** UV/vis absorption of **Ru-2** in the absence and presence of (A) GSH or (B) BSA. All spectra were acquired 12 h after GSH or BSA addition at 37 °C in PBS buffer (pH 7.4).

**Table S1.** Intracellular content of **Ru-1***^a^* and **Ru-2** *^a^* were determined by ICP-MS at different times and concentration.

| T_R_(hours)/  Concentration(μM) | Cellular-**Ru-1** | Cellular-**Ru-2** | Cellular-**Ru-1** | Cellular-**Ru-2** | Cellular-**Ru-1** | Cellular-**Ru-2** | Cellular-**Ru-1** | Cellular-**Ru-2** |
| --- | --- | --- | --- | --- | --- | --- | --- | --- |
|  | 5 | 5 | 10 | 10 | 15 | 15 | 20 | 20 |
| 1 | 120.3±9.5 | 85.4±9.3 | 146.3±13.7 | 99.4±9.6 | 178.5±15.4 | 115.8±11.7 | 215.9±19.4 | 138.7±15.3 |
| 3 | 168.5±13.0 | 112.4±9.5 | 216.8±16.9 | 134.6±8.3 | 246.7±25.4 | 159.4±8.5 | 312.4±21.5 | 181.3±9.3 |
| 6 | 241.2±10.6 | 142.5±9.6 | 299.4±1.2 | 173.2±8.9 | 343.8±17.0 | 204.6±8.9 | 432.5±12.4 | 235.2±16.6 |

*^a^*ng Ru/10^6^ A549 cell number.
